# Transcription factor VdCmr1 is required for pigment production, protection from UV irradiation, and regulates expression of melanin biosynthetic genes in *Verticillium dahliae*

**DOI:** 10.1099/mic.0.000633

**Published:** 2018-02-27

**Authors:** Yonglin Wang, Xiaoping Hu, Yulin Fang, Amy Anchieta, Polly H. Goldman, Gustavo Hernandez, Steven J. Klosterman

**Affiliations:** ^1^​ College of Forestry, Beijing Forestry University, Beijing, PR China; ^2^​ Department of Plant Pathology, College of Plant Protection, Northwest A&F University, Yangling, PR China; ^3^​ United States Department of Agriculture, Agricultural Research Service, 1636 E. Alisal St., Salinas, CA 93905, USA

**Keywords:** DHN, melanin, fungi, pigment, regulation, pathogenicity

## Abstract

*Verticillium dahliae* is a soilborne fungus that causes vascular wilt diseases on numerous plant species worldwide. The production of darkly melanized microsclerotia is crucial in the disease cycle of *V. dahliae*, as these structures allow for long-term survival in soil. Previously, transcriptomic and genomic analysis identified a cluster of genes in *V. dahliae* that encodes some dihydroxynaphthalene (DHN) melanin biosynthetic pathway homologues found in related fungi. In this study, we explored the roles of cluster-specific transcription factor VdCmr1, as well as two other genes within the cluster encoding a polyketide synthase (VdPKS1) and a laccase (*VdLac1*), enzymes at initial and endpoint steps in DHN melanin production. The results revealed that *VdCmr1* and *VdPKS1* are required for melanin production, but neither is required for microsclerotia production. None of the three genes were required for pathogenesis on tobacco and lettuce. Exposure of Δ*VdCmr1* and wild-type strains to UV irradiation, or to high temperature (40 °C), revealed an approx. 50 % reduction of survival in the Δ*VdCmr1* strain, relative to the wild-type strain, in response to either condition. Expression profiles revealed that expression of some melanin biosynthetic genes are in part dependent on *VdCmr1*. Combined data indicate VdCmr1 is a key regulator of melanin biosynthesis, and that via regulation of melanogenesis, VdCmr1 affects survival of *V. dahliae* in response to abiotic threats. We conclude with a model showing regulation of *VdCmr1* by a high osmolarity glycerol response (Hog)-type MAP kinase pathway.

## Introduction

The fungus *Verticillium dahliae* causes economically important vascular wilt diseases on more than 200 plant species worldwide [1]. The list of plant species affected by *V. dahliae* is continually expanding as new hosts are identiﬁed [2]. *V. dahliae* is difficult to control, owing in part to the production of melanized resting structures, known as microsclerotia, which can survive for years in the soil [3].

Fungal dihydroxynaphthalene (DHN) melanin is composed of polymerized phenolic or indolic compounds, and its biosynthesis was originally discovered in *V. dahliae* [4]. DHN-melanin biosynthesis begins with a polyketide synthase (PKS) that catalyses the conversion of acetyl-CoA to 1,3,6,8-tetrahydroxynaphthalene (1,3,6,8-THN). Through a series of downstream reactions catalysed by reductases and dehydratases, 1,8-DHN is produced [4–6].

Melanin has a protective role against UV irradiation in the microsclerotia of *V. dahliae*, and melanin deposition may further provide protection against temperature extremes, enzymatic lysis, nutrient deprivation and fungicidal activities [4]. The appearance of melanin is tightly coupled with latter stages of microsclerotial development in *V. dahliae*, and its very appearance implies microsclerotia maturation [7, 8]. Thus, melanin is vitally important to the production of functional microsclerotia. Understanding the genetics and molecular mechanisms that regulate melanin biosynthesis can expand our insights into microsclerotia formation and may lead to novel control measures to counter the threat of Verticillium wilt. Mutants of melanin biosynthesis have been characterized to some extent in *V. dahliae* [9, 10], including recent studies on molecular genetic analyses of *Vayg1*, a putative enzyme-encoding gene that catalyses an initial step of the melanin biosynthesis [11] and *VdPKS1*, a PKS necessary for melanin production [12]. However, we lack a thorough understanding of the genetic network that regulates melanin biosynthesis in *V. dahliae*, especially from a putative cluster of melanogenesis-related genes previously identified [7].

The availability of the *V. dahliae* genome has facilitated genome-scale investigations [13], including those examining the molecular genetic basis to melanin biosynthesis [7, 8, 11, 12]. Analyses of the genomic sequence of *V. dahliae* have revealed highly homologous gene sets that are known to contribute directly to melanin biosynthesis in other fungi, in addition to orthologues of other signalling components necessary for melanin biosynthesis. Prominent among the latter findings, mitogen-activated protein kinases (MAPKs) control melanin biosynthesis in *V. dahliae*. Orthologues of the high osmolarity glycerol (HOG) response MAPK pathway, including VdHog1 [14], VdPbs2 [15] and VdMsb [16], are all involved in melanin biosynthesis signalling since each of the gene replacement mutants for each of these strains is melanin deficient, and known melanin biosynthetic gene orthologues were substantially downregulated in VdHog1 and VdPbs2 mutants. Several transcription factors were recently shown to regulate melanin biosynthesis, such as VdMcm1 [17], VdCrz1 [18] and Vta2 [19]. These studies have strengthened understanding of the regulation of melanin biosynthesis in *V. dahliae*. However, with the exception of the PKS encoding gene, *VdPKS1* [12], genes that directly participate in melanin biosynthesis have not been systematically characterized in *V. dahliae* [13].

Expression of melanin biosynthetic genes in fungi is regulated in part by Cmr1 type transcription factor orthologues, found within secondary metabolism clusters of genes in some plant pathogenic fungi, such as *Magnaporthe oryzea*, *Cochliobolus heterostrophus, Alternaria brassicicola* and *Botrytis cinerea* [20–25]. The role of Cmr1 is important for DHN melanin production, as mutants of *Cmr1* and its corresponding homologues typically lack pigmentation, or have an atypical colour [25], indicating that the function of Cmr1 homologues is conserved in fungi. Structurally, Cmr1 possesses two Cys_2_His_2_ zinc finger domains and one Zn(II)_2_Cys_6_ binuclear cluster domain, near its N-terminus, and this structure is also highly conserved.

Our previous transcriptomic studies of microsclerotia formation have shown that melanogenesis-associated genes, i.e. tetrahydroxynaphthalene reductase and scytalone dehydratase, were clearly upregulated in developing microsclerotia [7, 8]. Strikingly, genomic analysis of these differentially expressed melanogenesis-associated genes revealed a 48.8 kilobase-long cluster, which is believed to be a gene cluster in part responsible for melanin biosynthesis in *V. dahliae* [7]. Furthermore, a gene encoding a Cmr1 orthologue, an important regulator of melanin biosynthesis, is located within this cluster, and upregulated during microsclerotia formation as well. However, the role of the Cmr1 homologue and how this homologue may regulate gene expression to activate melanin biosynthesis, microsclerotia formation and pathogenicity have not been elucidated in *V. dahliae*. Mutation of one of the genes encoding a PKS (*VdPKS1*, *VDAG_00190*) was recently examined, revealing that this gene is required for melanin production in *V. dahliae,* and also for full virulence of the pathogen in cotton [12].

The objectives of the current study were to explore whether melanin biosynthesis and/or pathogenicity is dependent on *VdCmr1.* Secondary objectives included examination of whether VdCmr1 regulates gene expression of melanin-associated genes, and also whether two additional melanin biosynthetic homologue gene mutants characterized in this study are pathogenic. The results showed that *VdCmr1* is required for melanin biosynthesis and microsclerotia formation, and that VdCmr1 regulates expression of genes scattered throughout the genome of *V. dahliae* that are associated with melanin biosynthesis. We determined definitively that melanogenesis-associated genes in *V. dahliae*, including *VdCmr1*, *VdPKS1* and a laccase-encoding gene, *VdLac1*, are not required for pathogenicity on tobacco and lettuce. Based upon the current and previously published data, we propose a model regulatory pathway for melanin biosynthesis to further study melanin biosynthesis in relation to microsclerotia formation and pathogenicity.

## Methods

### *V. dahliae* strains and growth condition

The fungal strain VdLs.17 [26], originally isolated from lettuce, was used as the wild-type and recipient strain in this study to introduce genetic mutations. All strains were regularly cultured on potato dextrose agar (PDA) plates at 25 °C. Cultures were maintained long-term in closed vials on PDA, or as −80 °C stocks in 20 % glycerol. Antibiotic-resistant strains were grown on PDA amended with hygromycin B (25 µg ml^−1^) or geneticin (50 µg ml^−1^). Cultures were grown for 1 month before harvesting.

### Targeted gene deletions

For these studies, we used the Broad Institute *V. dahliae* gene identification numbers, and corresponding functional annotations and sequences [27]. The deletion constructs for the knockout of gene *VDAG_00189* (*VdLac1*) and *VDAG_00190* (*VdPKS1;* [12]) were used in *Agrobacterium tumefaciens*-mediated transformation (ATMT) of VdLs.17 to obtain independent mutant strains. For constructing *VdLac1*or *VdPKS1* gene deletions, a 5 µl BP clonase reaction included 15–20 ng of co-purified PCR flank products, 60 ng of pA-Hyg-OSCAR [28], 60 ng of pOSCAR and 1 µl BP clonase II enzyme mix (Invitrogen). The BP reaction was carried out at 25 °C for 16 h and terminated using 0.5 µl proteinase K (20 µg µl^−1^). This generated the final marker vector named as pOSCAR-190 or pOSCAR-189. The reaction mixture was then used to transform *E. coli* competent cells by standard heat shock methods. Plasmid was co-purified using the CWBIO EndoFree Plasmid Midi Kit (Cwbiotech) and digested with *Kpn*I and *Hind*III to verify deletion construct structure. ATMT procedures followed the method described [29]. Individual *V. dahliae* transformants were transferred to PDA plates supplemented with hygromycin B (50 µg ml^−1^) and 200 µM cefotaxime. Hygromycin B-resistant transformants were single-spore purified and screened by PCR amplification to identify gene-replacement mutants. Primer pairs were applied to identify gene-replacement mutants, with the hygromycin B resistance gene-specific primers and *VdLac1* and *VdPKS1* gene-specific primers (Table S1, available in the online version of this article).

To delete *VDAG_00195* (*VdCmr1*) in the genome of *V. dahliae*, a split-marker method was applied similar to that previously described [14]. First, approximately 1.5 kb of upstream (5′) and downstream (3′) ﬂanking sequences of *VdCmr1* were ampliﬁed with primer pairs PL62/PL695 and PL696/PL65, respectively. Then, the geneticin-resistance cassette was ampliﬁed with the Geneticinfor/Geneticinrev primers for deletion, which include approximately 20 bp that overlaps with the 5′ and 3′ ﬂanking sequences, respectively. The two deletion cassettes resulting from fusion PCR with primer pairs PL62/Geneticinrev and Geneticinfor/PL65 (Table S1) were used for protoplast transformation procedures following the method described by Wang *et al*. [14]. The transformants were selected on TB3 medium with 50 µg ml^−1^ geneticin.

Screening of all knockouts was initially performed with those primer sets for each gene listed in Table S1. The Southern blot analyses were performed (Figs S1 and S2) to conﬁrm the homologous recombination event of the three genes using a DIG High Prime DNA Labeling and Detection Starter kit according to the manufacturer’s instructions (Roche). Probes were labelled in PCR profiles of 95 °C for 2 min, followed by 40 cycles of 95 °C for 30 s, 60 °C 30 s, 72 °C 1 for min, with a final 7 min extension at 72 °C. The 50 µl reaction volumes included 5 µl of 10× PCR buffer (with MgCl_2_), 5 µl DIG labelling mix (diluted 0.6 µl 10× PCR DIG Probe synthesis mix in 20.5 µl 10× dNTP mix), 1 µl enzyme mix (Expand Hi Fidelity Taq, Roche), 250 pg plasmid containing the appropriate construct, and 200 nM of each primer. Restriction digestions of the DNA from mutant and wild-type strains were carried in 50 µl reaction volumes with 2 µl of the appropriate enzyme, and 5 µg DNA, unless indicated otherwise. DNA products were ethanol-precipitated and re-suspended in 20 µl of water, and loaded alongside 5 µl DIG-labeled DNA Molecular Weight Marker II (Roche) in a 0.8 % agarose gel, run at 60 V for 3.5 h. Gels were transferred to PVDF membrane (BioRad) overnight in 10× SSC, with 10 min depurination, 30 min denaturation and 30 min neutralization steps. Fig. S1(a) shows confirmation of the knockout of *VdCmr1*. Fig. S2 provides detail on the knockouts of genes *VdPKS1* and *VdLac1*.

**Fig. 1.**
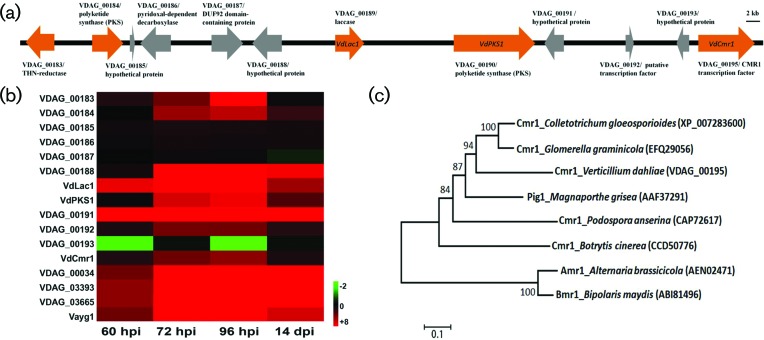
*VdCmr1* is located in a secondary metabolism cluster of genes in *V. dahliae,* and is upregulated during microsclerotia formation. (a) A 48.8 kilobase secondary metabolism cluster of genes in the *V. dahliae* genome. Black or orange arrows represent position and direction of individual genes. Orange represents genes that share homology with those having known roles in melanin biosynthesis in related fungi. The relative positions of *VdLac1*, *VdPKS1* and *VdCmr1* in this cluster are shown. (b) Expression data of the secondary metabolism gene cluster during microsclerotia formation. Four typical stages were represented during the entire process of microsclerotia formation at 60, 72, 96 h and at 14 days. Green represents the negative value of log2 (FPKM) and red represents the positive value of log2 (FPKM). Black represents the zero value of log2 (FPKM). (c) Phylogenetic tree of *VdCmr1* in *V. dahliae* and its homologues from other fungi using a neighbour-joining method in mega [40]. Accession numbers of VdCmr1 homologues are shown in parentheses. Bootstrap percentages over 50 % are indicated at the nodes.

The *VdCmr1* complemented strain was confirmed as shown by expression of a 191 bp cDNA product (Fig. S1b), and this complemented strain was referred to as VdCmr1_C. The *VdCmr1* sequence in this study was complemented with both the *VDAG_00194* plus the *VDAG_00195* sequence because homology searches and previous evidence indicated that these two sequences together encoded the single transcription factor *VdCmr1* (Fig. 1, Table 1 and Duressa *et al.* [7]). Additional sequences available [30] enabled the extension of primers into the 3′ flanking region of *VdCmr1* to obtain the full-length approx. 5.6 kb fragment used for complementation of strain VdCmr1 (Fig. S1b).

**Table 1.** Known and putative melanin biosynthetic genes of *V. dahliae*

Gene ID/putative function in V. dahliae*	**Name in *V. dahliae/*reference**	**Function†** **(orthologous fungal proteins)**	**References**
*VDAG_03665*/tetrahydroxynaphthalene reductase	……….	Potentially both tri- and tetra-hydroxynaphthalene reductase functions	[41, 42]
*VDAG_03393*	……….	Scytalone dehydratase	[43]
*VDAG_04954*/pigment biosynthesis protein Ayg1	*Vayg1/* [11]	Polyketide chain shortening	[44]
*VDAG_00190*/conidial yellow pigment biosynthesis PKS	*VdPKS1;* this study/ [12]	Polyketide synthase	[45]
*VDAG_00194/VDAG_00195*/transcription factor Pig1/CMR1	*VdCmr1;* this study	Transcription factor	[20–22, 25]
*VDAG_00189*/laccase	*VdLac1;* this study	Multicopper oxidase/laccase	[46]
*VDAG_00184/*amino acid adenylation‡	……….	Non-ribosomal polyketide synthase	[47]
*VDAG_00183/versicolorin reductase*	……….	Tetrahydroxynaphthalene reductase	[41]
*VDAG_05181*/tetrahydroxynaphthalene reductase§	……….	Tetrahydroxynaphthalene reductase	[41]
*VDAG_00034*/laccase	……….	Multicopper oxidase/laccase	[46]

*The gene ID and functional annotations of these genes in *V. dahliae* are derived from original Broad Institute annotations [27].

†Functions ascribed based upon known fungal orthologues.

‡The gene ORF is only partial as it was missanotated in the genome of VdLs.17.

§The putative tetrahydroxynaphthalene reductase encoding gene *VDAG_05181* resides in a previously described lineage specific region of VdLs.17 [27], and is presumably the result of a gene duplication specific to VdLs.17, and not in the core genomes of all *V. dahliae* strains.

### Exposure to UV irradiation and high and low temperatures

Conidial suspensions of each strain were spread onto potato dextrose agar (PDA; Fisher Scientific) amended with streptomycin (50 µg ml^−1^), kanamycin (50 µg ml^−1^) and tetracycline (12.5 µg ml^−1^) and incubated at 25 °C for 2 weeks. The fungus/agar mixture was removed from the plates, suspended in phosphate buffer (0.01 M pH 7.0) and blended in a Waring blender for three 10 s pulses. Conidia and very small hyphal and agar fragments were removed by filtering through Miracloth (22 µm pore size; Fisher Scientific). Material retained on the filter was re-suspended in phosphate buffer and sonicated with a Vibra-Cell probe sonicator (Sonics) for four 2 s pulses, followed by centrifugation for 5 min at 600 ***g***, which pelleted the microsclerotia below a layer of agar which was easily removed by pipetting. The microsclerotial pellets were then dried at room temperature and ground with a mortar and pestle, and filtered again with Miracloth (22 µm pore size). Microscopic examination revealed enrichment in microsclerotia. Liquid suspensions of the microsclerotia-enriched cultures were made in 0.01 M phosphate buffer (pH 7.0) and adjusted to approximately 1×10^6^ propagules ml^−1^ based on dilution plating. There were four replicates per strain, and each replicate was serially diluted in five 10-fold increments, and used in experiments.

For UV light treatments, 100 µl of inocula from each of the dilutions, prepared as described above, were plated onto NP-10 [31]. With lids removed, plates were placed in a biosafety cabinet (Labconco Purifier Logic+) under UV light (253 nm) for 3 h, after which the lids were replaced and the plates were incubated for 2 weeks at 25 °C. Colonies were enumerated and proportional germination was determined by comparing values obtained with the UV-irradiated cultures versus those without UV light treatment.

For low temperature treatment, nitrocellulose filters (Whatman 7141154) were soaked in 250 µl of inocula and dilutions, dried at room temperature and incubated at −20 °C for 7 days. The filters were placed on NP-10 and incubated for 2 weeks at 25 °C, after which the colonies were counted and relative germination determined as above.

For high temperature analysis, dilutions from each inoculum from each strain were plated onto NP-10, and incubated for 24 h at 40 °C. The plates were transferred to an incubator at 25 °C for 2 weeks, and proportional germination determined as above.

Proportional germination in the three treatments for fungal survival was determined by dividing c.f.u. ml^−1^ values obtained in each treatment replicate by those obtained following incubation of the same replicate dilutions at 25 °C with no treatment. These proportional values were log-transformed and analysed by ANOVA (JMP 12.2), with Tukey’s HSD used for mean comparisons. The two experiments were analysed separately, as there was a statistically significant (*P*<0.05) effect of experiment in all three of the parameters measured.

### Nucleic acid manipulation and TaqMan assays

Conidia were washed from membranes using 2 ml of distilled water and a cell spreader. The membranes containing hyphae were ground to a fine powder using liquid nitrogen in a mortar and pestle. An RNeasy Plant Mini kit (Qiagen, La Jolla, CA) was used to extract nucleic acids from 100 mg of the powder and the extraction steps included the 56 °C incubation and DNAse I (Qiagen, La Jolla, CA) on-column digestion. Extractions were further treated with TURBO DNase (Ambion, Austin, TX) at 37 °C for 30 min after extraction. RNA quality was checked using the Nanodrop (Thermo Scientific, Wilmington, DE) and quantified using a Qubit Fluorometer (Invitrogen, Carlsbad, CA). The RNA (250 ng) was reverse transcribed by denaturing at 65 °C with 0.5 µg Oligo d(T)_15_ and 0.77 mM dNTPs for 5 min, chilling on ice for at least 5 min and incubating at 55 °C for 45 min with 200 U SuperScript III (Invitrogen, Carlsbad, CA), 1X first strand synthesis buffer, 5 mM DTT and 40 U RNAsin (Promega, Madison, WI) in 20 µl reactions followed by 70 °C for 15 min to inactivate the reverse transcriptase. Four 20 µl reverse transcription reactions per sample were combined to use as a template. A volume of 2 µl cDNA was used as a template in 20 µl reactions containing: 1X Gene Expression Master Mix (ABI, Carlsbad, CA) and 1X Custom TaqMan Gene Expression Assay (900 nm primer, 200 nM taqman probe) (ABI, Carlsbad, CA) labelled with FAM and quenched with NFQ. The reaction profile included an initial 95 °C 10 min step followed by 40 cycles of 95 °C 15 s and 60 °C 30 s. Resulting quantification cycle values were analysed using REST 2009 software [32], enabling computation of statistical significance of relative expression analyses. Products were cloned into pCR 4.0-TOPO (Invitrogen) and sequenced to confirm primer specificity. The resulting plasmids were used in a five-step 10-fold dilution standard curve (5 ng to 500 pg) to test the efficiency of the TaqMan Gene Expression Assays. All assays were >90 % efficient. There were two biological replicates per treatment, and each sample collected was run in triplicate in single probe reactions.

### Pathogenicity assay

For pathogenicity assays, conidia were harvested from 15-day-old cultures grown in liquid CM by ﬁltration through two layers of Miracloth and resuspended at 10^6^ conidia/ml in sterile distilled water. For each experiment, 10 two-week-old *Nicotiana benthamiana* or 10 two-week-old lettuce plants were inoculated with each of the *V. dahliae* genotypes by dipping the roots for 5 min in inoculum, and transferring the plants into potting soil in cups in a greenhouse. There were two replicate treatments, with each treatment containing 20 plants. The height of tobacco seedlings was measured at 45 dpi. Vascular discolouration symptoms of lettuce seedlings inoculated with the above strains were observed by cutting the roots longitudinally 10 weeks after inoculation.

## Results

### Identification of VdCmr1

We have previously created several transcriptomic databases to elucidate molecular processes of microsclerotia biogenesis and melanin synthesis in *V. dahliae* [7, 8, 17]. The timing of melanin production relative to microsclerotial development and in multiple expression libraries was previously characterized by Xiong *et al.* [8]. Analysis of these libraries revealed that a secondary metabolism gene cluster encodes some melanin biosynthetic gene homologues that are upregulated during microsclerotia formation. The cluster spans a 48.8 kb region in the *V. dahliae* genome and contains melanogenesis-related enzyme-encoding genes, such as THN reductases (*VDAG_00183; VDAG_03665*), PKSs (*VDAG_00190; VdP KS1*) and a laccase (*VDAG_00189; VdLac1*) (Fig. 1a). Some of genes were likely misassembled, such as *VDAG_00184* that appears to be a truncated PKS (Table 1). In addition, the expression profile of this cluster of genes during microsclerotia development showed that the melanogenesis-related genes were upregulated in microsclerotia-producing cultures (Fig. 1b). Examination of this cluster in detail revealed that this cluster comprises two transcription factor encoding genes (*VDAG_00192* and *VDAG_00195; VdCmr1*). *VDAG_00192* encodes a putative transcription factor, while *VdCmr1* encodes a homologue of the transcription factor CMR1 previously reported to be involved in fungal melanin synthesis [20, 21]. Phylogenetic analysis revealed that VdCmr1 exhibited a high degree of amino acid sequence similarity to those CMR1 orthologues from other fungi (Fig. 1c).

### Functional analyses of *VdCmr1* and two additional genes of a putative melanin biosynthetic gene cluster in *V. dahliae*

To examine the function of genes in the putative melanin biosynthetic gene cluster in *V. dahliae*, gene deletion mutants were prepared for several key genes through homologous recombination and replacement with a gene conferring hygromycin B resistance (for *VdLac1* and *VdPKS1*) or geneticin resistance (for *VdCmr1*). Screening of the transformants by PCR using gene-specific primers (Table S1) and confirmation of the knockouts by Southern blot revealed that the three genes were successfully replaced (Figs S1 and S2). The *VdLac1* deletion mutant (*VdLac1-*5KO), ectopic strain (*VdLac1*-7Ect), *VdPKS1* deletion mutant (*VdPKS1*-7KO), ectopic strain (*VdPKS1*-14Ect), *VdCmr1* deletion mutant (*VdCmr1*-9KO) and ectopic strain (*VdCmr1*-11Ect) were selected for further comparisons with the wild-type strain VdLs17.

**Fig. 2.**
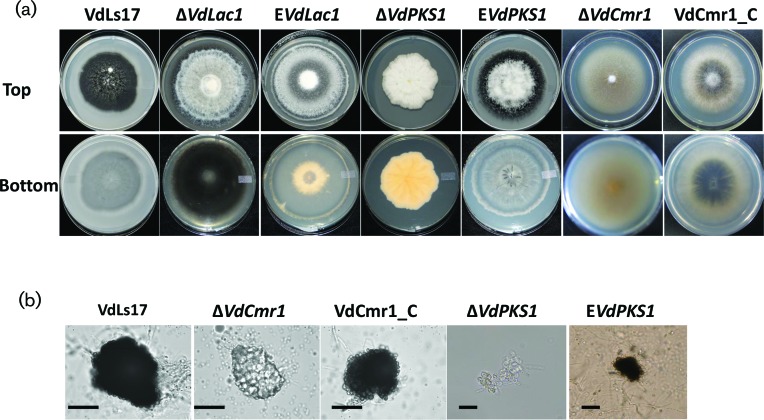
Effect of three gene deletions on melanin production and colony morphology in *V. dahliae*. (a) Colony appearance of *V. dahliae* wild-type strain (VdLs17), *VdLac1* deletion mutant (Δ*VdLac1*) and ectopic strain E*VdLac1 VdPKS1* deletion mutant (Δ*VdPKS1*) and ectopic strain (E*VdPKS1*), and *VdCmr1* deletion mutant (Δ*VdCmr1*), ectopic strain (E*VdCmr1*) and the Δ*VdCmr1*-complemented strain (VdCmr1_C) on PDA medium after a 2 week incubation at 25 °C with top and bottom views. (b) Examination of the microsclerotia produced by the wild-type strain VdLs.17 relative to the microsclerotia produced by the corresponding gene knockout strains for *VdCmr1* and *VdPKS1.* The restoration of pigment production is observed for strain VdCmr1_C. Scale bars=20 µm.

All mutants showed normal growth rate and morphology, as examined by microscopy for conidiophore structure and conidia. However, both *VdPKS1* and *VdCmr1* deletion mutants failed to melanize in culture while the wild-type strain VdLs.17 and the *VdLac1* deletion mutant formed melanized microsclerotia (Fig. 2a). Therefore, *VdPKS1* and *VdCmr1* are essential for appropriate melanization in *V. dahliae*, but not *VdLac1*. Microscopic analyses revealed that both *VdPKS1* and *VdCmr1* deletion mutants produced microsclerotia, but these microsclerotia were devoid of the dark melanization observed in the microsclerotia of the wild-type VdLs.17 strain, or either of the ectopic insertion strains (Fig. 2b).

### VdCmr1 regulates the expression of genes involved in melanin biosynthesis

**Fig. 3.**
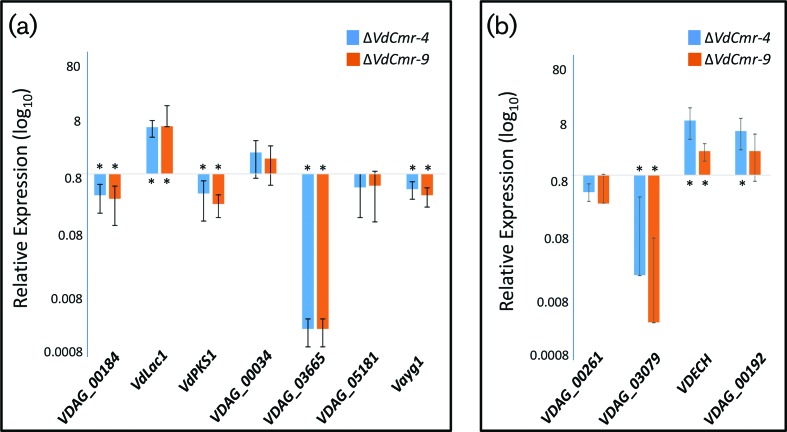
Transcriptional analysis of melanogenesis and non-melanogenesis associated genes in the *VdCmr1* deletion mutants in *V. dahliae.* (a) Relative expression levels of melanogenesis-associated genes and (b) non-melanogenesis-related genes including genes differentially expressed in microsclerotial development [7], but having roles thought to be independent of melanin production such as *VDAG_00261* (IDI3 homologue), *VDAG_03079* (catalase), *VDECH* (endochitinase; *VDAG_08741* [33]). The experiments were carried out using two independent *VdCmr1* deletion mutants (designated −4KO and −9KO) and were compared with wild-type strain (VdLs17). The ubiquitin gene was used as an internal reference. The error bars represent standard deviations. There were two biological experiments performed for each mutant strain, Δ*VdCmr-4* and Δ*VdCmr-9*, and three technical replicates per biological experiment. Asterisks (_*_) indicate that the fold changes were significant.

Since VdCmr1 is a homologue of the fungal CMR1 transcription factors, we investigated the impact of VdCmr1 on the transcription of melanin-associated genes in *V. dahliae* by relative expression analyses in TaqMan assays. The expression level of genes associated with DHN melanin biosynthesis located in the cluster revealed that transcripts of the PKS-encoding genes *VDAG_00184* and *VdPKS1* (*VDAG_00190*) were significantly (*P*<0.05) reduced in the *VdCmr1* deletion mutants. Transcripts of *VdLac1* were upregulated in the mutants, relative to the levels observed in the wild-type VdLs.17 strain (Fig. 3a), suggesting VdCmr1 upregulates *VDAG_00184* and *VdPKS1*, and inhibits expression of *VdLac1*. The expression level of the transcription factor encoding gene *VDAG_00192*, within the same gene cluster, was not significantly up or downregulated in both *VdCmr1* deletion mutants (Fig. 3a). Relative expression levels of additional homologues of melanin biosynthetic-related genes *VDAG_00034* (laccase)*, VDAG_03665* (scytalone dehydratase)*, VDAG_04954* (PKS Vayg1)*, VDAG_05181* (THN reductase), were analysed in two *VDAG_00195* deletion mutants (Fig. 3a). Among these genes, *VDAG_00034* (laccase) and *VDAG_05181* (THN reductase) were not differentially expressed (*P*<0.05) in the *VdCmr1* deletion mutant background (Fig. 3b). The THN-reductase-encoding gene VDAG_03665 was over 100-fold reduced in expression in the VdCmr1 deletion mutants (Fig. 3b). Separate replicate TaqMan assays revealed that *VDAG_00183,* encoding a THN reductase, was also not differentially expressed in the *VdCmr1* deletion mutant strains (data not shown).

We also examined a small subset of genes differentially expressed in microsclerotial development [7], but which are thought to have roles independent of melanin production such as *VDAG_00261* (encoding an IDI-3 homologue), and *VDAG_03079* (catalase). *VDAG_08741* encoding the endochitinase VDECH [33] was significantly (*P*<0.05) upregulated in the *VdCmr1* deletion mutants (Fig. 3b), suggestive of inhibition during microsclerotial development, corroborating previous findings [7].

Together, these expression data suggest that *VdCmr1* controls gene expression necessary for melanin biosynthesis in *V. dahliae,* and also the expression of two of those genes implicated in microsclerotia development.

### *VdPKS1*, *VdLac1* and *VdCmr1* are dispensable for pathogenicity

**Fig. 4.**
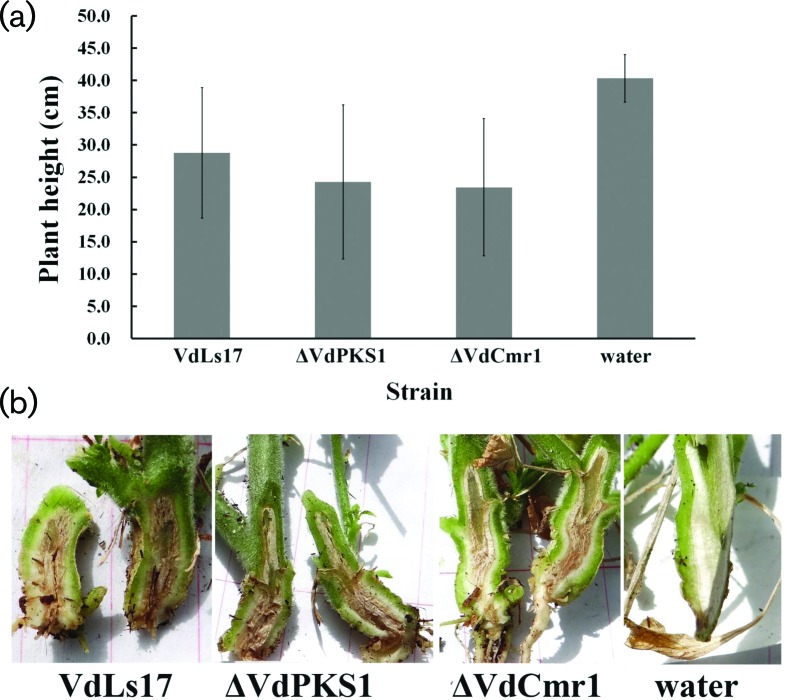
Pathogenicity analyses of *VdPKS1* and *VdCmr1* gene deletion mutants of *V. dahliae*. (a) The height of tobacco incubated with wild-type strain VdLs17, the *VdPKS1* deletion mutant, the *VdCmr1* deletion mutant, or mock-inoculated with water and examined at 45 dpi. (b) Vascular discoloration symptoms of tobacco seedlings inoculated with the above strains were observed by longitudinal sectioning of the crown region and lower stem.

We also assayed two of the deletion mutants prepared in this study in plant pathogenicity assays using lettuce and tobacco. On tobacco, *VdCmr1* and *VdPKS1* mutant strains were as virulent as the wild-type strain VdLs.17, as was evident in the comparisons of plant height (Fig. 4a). As compared to the mock-inoculated plants, those plants inoculated with either the Δ*VdPKS1*, Δ*VdCmr1* or wild-type strain VdLs.17, showed clear wilt symptoms on tobacco (Fig. 4) and lettuce (Fig. S3). Mutant and VdLs.17-inoculated plants also showed indistinguishable levels of stem vascular discolouration in tobacco (Fig. 4b). Thus, genes *VdPKS1* and *VdCmr1*, while critical to melanogenesis, play no role in pathogenesis in tobacco or lettuce, in strain VdLs.17.

### Effect of UV irradiation and high and low temperature on the survival of *VdPKS1* and *VdCmr1* mutants

**Fig. 5.**
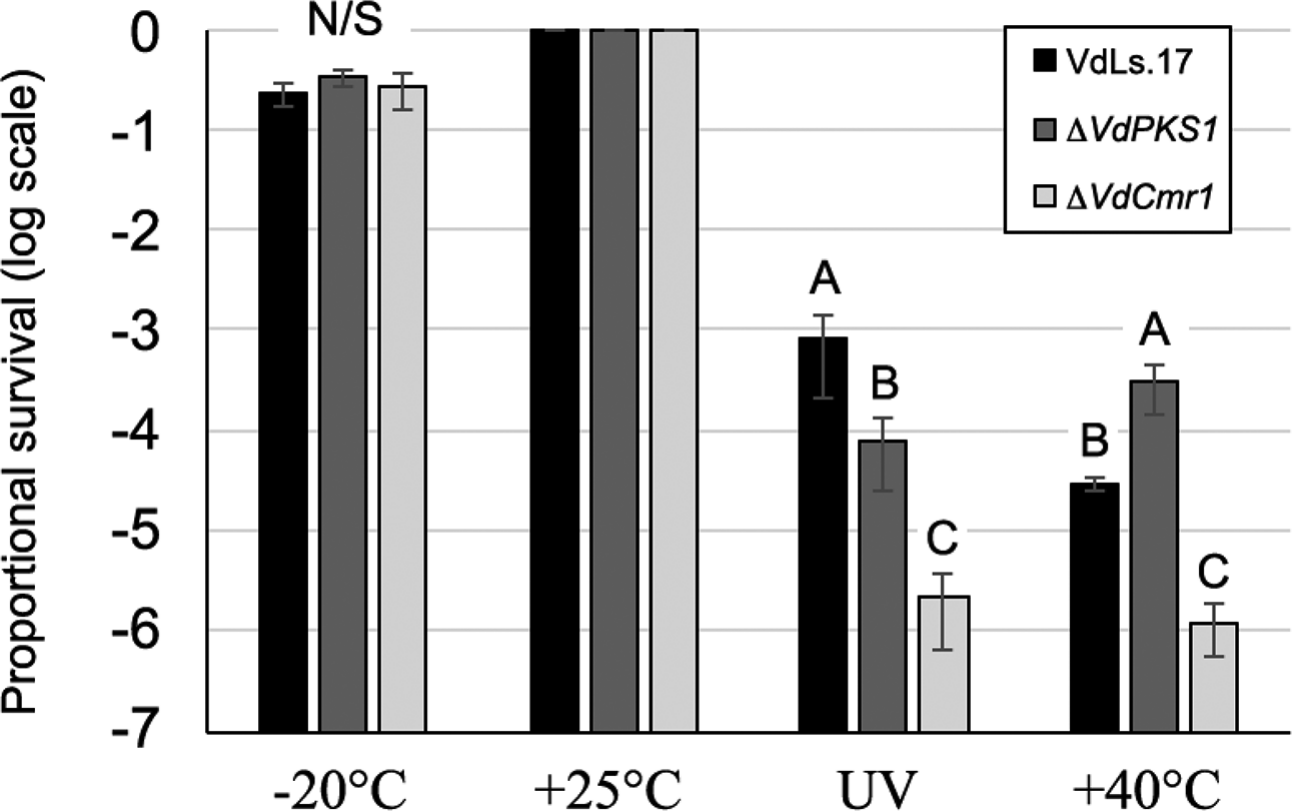
Survival of *V. dahliae* wild-type strain Ls.17 and the non-melanin-producing strains Δ*VdPKS1* and Δ*VdCmr1* under four treatment regimes. Values shown are in proportion of c.f.u. relative to the 25 °C treatment. Different letters associated with each bar reflect significantly different (*P*<0.05) values within the same treatment. N/S=differences not significant. There were two biological replicates, and four technical replicates for each treatment.

There were no significant differences in survival (as determined by c.f.u. measurements) among the different strains examined in response to low temperature incubation (*P*=0.53 and *P*=0.18 for experiments 1 and 2, respectively) (Fig. 5).

In response to UV irradiation, a significant (*P*<0.0001) reduction in survival was observed in both the Δ*VdPKS1* and Δ*VdCmr1* strains in comparison to the wild-type strain (Fig. 5).

In response to high temperature, the Δ*VdCmr1* strain showed significantly (*P*<0.0001) lower survival than the wild-type strain in response to high temperature incubation. The Δ*VdPKS1* strain exhibited significantly (*P*<0.0001) higher survival as compared with the wild-type strain following this treatment (Fig. 5).

### Model of the genetic regulation of VdCmr1-dependent melanin production

**Fig. 6.**
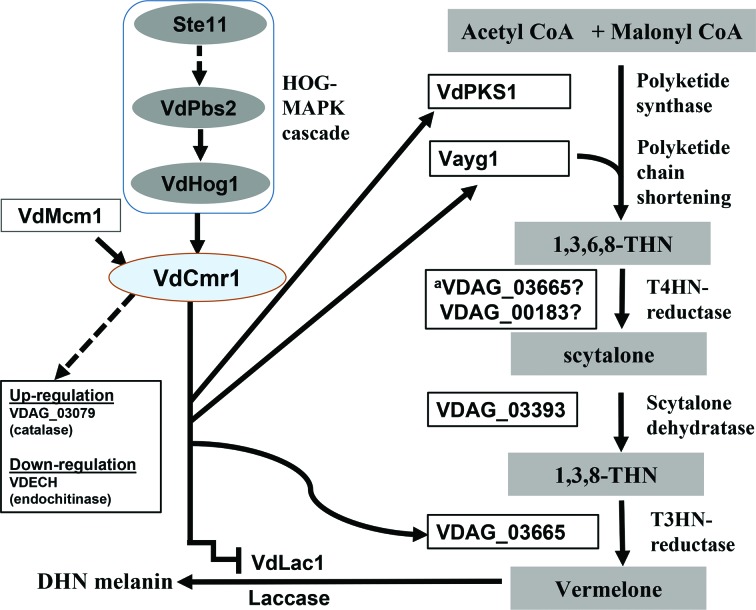
Schematic diagram depicting VdCmr1-dependent DHN melanogenesis in *V. dahliae.* Key proteins involving the DHN melanogenesis pathway are depicted from the initial acetyl-CoA or malonyl-CoA precursors through production of DHN melanin [4]. The Ssk2-Pbs2-Hog1 signalling cascade is a regulator of melanin biosynthesis in *V. dahliae* [14]. The transcription factor VdMcm1 and the polyketide chain shortening enzyme Vayg1 also modulate DHN melanogenesis. Our work identified several key genes that contribute to DHN melanin biosynthesis and that are regulated by VdCmr1. Some of these genes are found within the cluster enriched in DHN melanin biosynthetic genes within the *V. dahliae* genome. The non-melanin synthesis-related genes, up or downregulated during microsclerotial development [7], are regulated by VdCmr1 as demonstrated in this study; these genes *VDAG_03079* (catalase) and *VDAG_08741 VDECH* (endochitinase, *VDAG_08741*) also reside outside of the gene cluster enriched in melanin biosynthetic genes. ^a^VDAG_03665 may catalyse both T4HN and T3HN reductase steps based upon the strong homology of VDAG_03665 to the THR1 protein of *Colletotrichum lagenarium,* which can catalyse both T4HN and T3HN reductase steps [41].

We explored the regulation of gene expression dependent on VdCmr1 within the putative melanin biosynthetic cluster of genes in *V. dahliae*, and also some that were expressed at higher levels in microsclerotia production [7], and outside of the cluster. Combined with our previous results of regulation of genes involved in melanin production [14–16], we propose that VdCmr1 acts as a general regulator of melanin biosynthesis and the cluster of encoded proteins in which it resides is a major contributor to melanin biosynthesis. Previously, several upstream regulators of VdCmr1 have been identified, including the HOG signalling pathway (Ssk2-Pbs-Hog1 cascade), MADS-box transcription factor VdMcm1 [17] and Vayg1 [11]. Transcriptional analysis of genes involved in melanin biosynthesis revealed that VdCmr1 regulates expression of genes located in this cluster which contributes to melanin production, while also controlling expression of genes at other locations in the genome, some of which may or may not be involved in melanogenesis. Some of these genes were identified previously as differentially expressed during microsclerotia production, and thought to be important for development (Fig. 6). In summary, our results support a genetic network for melanin production in *V. dahliae* via VdCmr1, in which the Ssk2-Pbs-Hog1 cascade, VdMcm1, Vayg1 and unknown regulators activate VdCmr1 to control transcriptional and post-transcriptional processes of melanin biosynthesis and microsclerotial development in *V. dahliae*.

## Discussion

Melanized microsclerotia formation is critically important in the life cycle and disease spread of *V. dahliae* [2]. The aim of the current study was to examine the function of the homologue of fungal CMR1, located within a 48.4 kb gene cluster that encodes several other melanogenesis-related genes identified previously in *V. dahliae* [7, 8]. Based on related DHN melanin biosynthetic pathways in fungi, the PKS enzyme is one of the initial enzymes of the pathway, while the laccase is thought to be a terminal enzyme, acting at the last step of DHN melanin production. The *VdCmr1* transcription factor and two other genes (*VdLac1* and *VdPKS1*) were deleted by homologous recombination and gene replacement and provided analyses of the phenotypes of these mutants in culture, and in pathogenicity assays. Collectively, our study expands the understanding of the genetics of melanin biosynthesis, its regulation and its relationship to virulence in *V. dahliae*.

DHN melanin is a ubiquitous pigment in the fungal kingdom. A transcription factor involved in DHN melanin biosynthesis, known as CMR or BMR, has been characterized in several fungi, such as *M. oryzae* [22], *C. heterostrophus* [21], *A. brassicicola* [23] and *B. cinerea* [24]. Loss of function of CMR1 homologues, or some of their downstream affected genes in these fungi, typically results in melanin deficiency. We identified *VdCmr1* in this study, as a regulator of melanin biosynthesis in *V. dahliae*. In addition, four melanin biosynthesis-associated gene homologues are located together in a 48.4 kb region in strain VdLs.17, and their organization in the cluster is similar to that observed in *C. heterostrophus* [21]. When *VdLac1* and *VdPKS1* were separately deleted in this cluster in *V. dahliae*, we found that the *VdPKS1* was required for melanin production, but not the laccase. Similarly, Zhang *et al.* [12] also found that *VdPKS1* was required for pigment production in a cotton isolate of *V. dahliae*. With regard to the laccase, a close laccase paralogue encoded by *VDAG_00034* is outside of the melanin gene cluster, and potentially has an overlapping function that compensated for the *VdLac1* mutation in this study. However, as shown in this study, *VDAG_00034* was not significantly differentially expressed in the *VdCmr1* mutant strains, suggesting that this gene is not under regulatory control of VdCmr1.

The ﬁrst enzyme in the DHN melanin biosynthesis pathway is PKS. The putative gene cluster containing *VdCmr1* also encodes two PKS genes (*VDAG_00184* and *VdPKS1*; Table 1). The cluster also contains genes encoding other enzymes involved in melanin biosynthesis, such as THN reductase (*VDAG_00183*) and laccase (*VdLac1*). VdCmr1 regulates transcription of PKS, laccase, THN reductase-encoding genes. We speculate that *V. dahliae* employs a melanin biosynthetic pathway encoded in part by the 48.8 kb cluster of the secondary metabolism-related genes shown in Fig. 1, and those genes scattered throughout the genome, allowing for their individual regulation by VdCmr1. Intriguingly, we also noted that VdCmr1 regulates the expression of two genes scattered throughout the genome that were previously characterized as up or downregulated in microsclerotia-producing cultures of *V. dahliae*. Since microsclerotial development and melanin production are tightly linked, as previously observed [7, 8], VdCmr1 may act as a master regulator that controls non-essential aspects of microsclerotial development (Fig. 6), to appropriately coordinate the inclusion of DHN melanin at the later stages of microsclerotial maturation.

The relationship between melanin and virulence in fungi seems at times paradoxical. That is, melanin-deficient mutants are commonly identified as non-pathogenic or as reduced in virulence, not only in *V. dahliae*, but also in other fungi. In this study, pathogenicity assays showed that melanin-deficiency was not associated with reduced virulence as has been reported for the APSES transcription factor *Vst1* mutant of *V. dahliae* [34], or related mutants in other fungi [21]. Many studies have revealed a linkage between *V. dahliae* melanin-deﬁcient strains and reduced virulence or loss of pathogenicity [11, 14, 15, 18] but often many of these types of mutants compromised in melanin production are also defective in microsclerotia production [13]. Based on these accumulated findings, we speculate that DHN melanin alone produced through a VdCmr1-dependent pathway is not required for pathogenicity in *V. dahliae*. Interestingly, in support of this conclusion, the *VdCmr1* homologue *Amr1* actually suppresses virulence in *Alternaria brassicicola* [23].

The recent work of Zhang *et al*. [12] showed that deletion of *VdPKS1* resulted in a slight but significant reduction in virulence in the *V. dahliae* cotton isolate V592, although the *VdPKS1* mutant nevertheless caused disease symptoms on cotton. Since the mutant was produced in the cotton isolate V592, it is difficult to directly compare the results of the current study with those obtained by Zhang *et al*. [12]. Some genetic mutants of non-melanogenesis related genes in *V. dahliae* show reduced virulence on one host, but not on another host [27]. It is clear, however, that some pigment mutants of *V. dahliae* can also be compromised in virulence. For example, *Vayg1*, encoding the homologue of fungal enzymes involved in polyketide chain shortening to 1, 3, 6, 8-THN (Table 1), is necessary for melanin and microsclerotia production, and for full virulence in *V. dahliae* [11].

Many studies support a role of fungal DHN melanin in protection from abiotic stresses such as UV irradiation and temperature extremes [35–38]. This current study provides further direct evidence of the important role of DHN melanin in the protection of *V. dahliae* from UV irradiation. In this study, both the Δ*VdPKS1* and Δ*VdCmr1* strains were compromised in survival rate following UV irradiation, relative to the wild-type strain. Moreover, the Δ*VdCmr1* strain exhibited a decreased survival rate following exposure to high temperature. However, the *VdPKS1* mutant strain showed increased survival relative to the wild-type strain VdLs.17. The increased survival of the Δ*VdPKS1* strain at the high temperature (60 °C) examined is difficult to explain, but may be due to a pleiotropic effect, as suppressor mutations for temperature sensitivity affecting cell wall biogenesis have been identified [39]. Nonetheless, both the Δ*VdPKS1* and Δ*VdCmr1* strains, which are compromised in melanin production, showed significant reductions in their survival rates following UV irradiation.

Accumulating evidence indicates MAPK cascades are tightly coupled to the activation of VdCmr1. The HOG signalling pathway positively regulates *VdCmr1* [14, 15]. Phenotypes of *VdHog1* and *VdPbs2* kinase deletion mutants, which are similar to those of the *VdCmr1* mutant suggest that VdCmr1 may be a novel downstream regulator of a HOG-like pathway that controls melanin biosynthesis. However, it is unclear how VdHog1 interacts with VdCmr1 at this time. Intriguingly, other genes such as *VdMcm1* and *Vayg1* also regulate expression of *VdCmr1* and are also important for full virulence [11, 17]. Hence, there appears to be a complex interplay between melanin production and virulence in fungi. Taken together our results provide evidence of the association of VdCmr1-dependent melanin production and signalling pathways in *V. dahliae*.

We have demonstrated that while melanin production is *VdCmr1*-dependent in *V. dahliae*, *VdCmr1* is clearly not required for microsclerotia production and pathogenicity on tobacco and lettuce. The VdCmr1 transcription factor also controls the expression of a number of melanin biosynthesis-associated genes. As a critical regulator of melanin production, *VdCmr1* may integrate different signalling pathways to balance melanin production and development. The data presented in this study will facilitate future evaluations of the melanin biosynthetic pathway and development in *V. dahliae*.

## Supplementary Data

377 Supplementary File 1

## References

[1] Pegg G, Brady B (2002). Verticilliumwilts.

[2] Klosterman SJ, Atallah ZK, Vallad GE, Subbarao KV (2009). Diversity, pathogenicity, and management of *Verticillium* species. Annu Rev Phytopathol.

[3] Wilhelm S (1955). Longevity of the Verticillium wilt fungus in the laboratory and field. Phytopathology.

[4] Bell AA, Wheeler MH (1986). Biosynthesis and functions of fungal melanins. Annu Rev Phytopathol.

[5] Chumley FG (1990). Genetic analysis of melanin-deficient, nonpathogenic mutants of *Magnaporthe grisea*. Mol Plant-Microbe Interact.

[6] Stipanovic RD, Bell AA (1976). Pentaketide metabolites of *Verticillium dahliae*. 3. Identification of (-)-3,4-dihydro-3,8-dihydroxy-1(2h)-naphtalenone((-)-vermelone) as a precursor to melanin. J Org Chem.

[7] Duressa D, Anchieta A, Chen D, Klimes A, Garcia-Pedrajas MD (2013). RNA-seq analyses of gene expression in the microsclerotia of *Verticillium dahliae*. BMC Genomics.

[8] Xiong D, Wang Y, Ma J, Klosterman SJ, Xiao S (2014). Deep mRNA sequencing reveals stage-specific transcriptome alterations during microsclerotia development in the smoke tree vascular wilt pathogen, *Verticillium dahliae*. BMC Genomics.

[9] Bell AA, Puhalla JE, Tolmsoff WJ, Stipanovic RD (1976). Use of mutants to establish (+)-scytalone as an intermediate in melanin biosynthesis by *Verticillium dahliae*. Can J Microbiol.

[10] Stipanovic RD, Bell AA (1977). Pentaketide metabolites of *Verticillium dahliae*. II. Accumulation of naphthol derivatives by the aberrant-melanin mutant BRM-2. Mycologia.

[11] Fan R, Klosterman SJ, Wang C, Subbarao KV, Xu X (2017). *Vayg1* is required for microsclerotium formation and melanin production in *Verticillium dahliae*. Fungal Genet Biol.

[12] Zhang T, Zhang B, Hua C, Meng P, Wang S (2017). *VdPKS1* is required for melanin formation and virulence in a cotton wilt pathogen *Verticillium dahliae*. Sci China Life Sci.

[13] Klimes A, Dobinson KF, Thomma BP, Klosterman SJ (2015). Genomics spurs rapid advances in our understanding of the biology of vascular wilt pathogens in the genus *Verticillium*. Annu Rev Phytopathol.

[14] Wang Y, Tian L, Xiong D, Klosterman SJ, Xiao S (2016). The mitogen-activated protein kinase gene, *VdHog1*, regulates osmotic stress response, microsclerotia formation and virulence in *Verticillium dahliae*. Fungal Genet Biol.

[15] Tian L, Wang Y, Yu J, Xiong D, Zhao H (2016). The mitogen-activated protein kinase kinase VdPbs2 of*Verticillium dahliae* regulates microsclerotia formation, stress response, and plant infection. Front Microbiol.

[16] Tian L, Xu J, Zhou L, Guo W (2014). VdMsb regulates virulence and microsclerotia production in the fungal plant pathogen *Verticillium dahliae*. Gene.

[17] Xiong D, Wang Y, Tian L, Tian C (2016). MADS-Box transcription factor VdMcm1 regulates conidiation, microsclerotia formation, pathogenicity, and secondary metabolism of *Verticillium dahliae*. Front Microbiol.

[18] Xiong D, Wang Y, Tang C, Fang Y, Zou J (2015). VdCrz1 is involved in microsclerotia formation and required for full virulence in *Verticillium dahliae*. Fungal Genet Biol.

[19] Tran VT, Braus-Stromeyer SA, Kusch H, Reusche M, Kaever A (2014). Verticillium transcription activator of adhesion Vta2 suppresses microsclerotia formation and is required for systemic infection of plant roots. New Phytol.

[20] Tsuji G, Kenmochi Y, Takano Y, Sweigard J, Farrall L (2000). Novel fungal transcriptional activators, Cmr1p of Colletotrichum lagenarium and pig1p of *Magnaporthe grisea*, contain Cys_2_His_2_ zinc finger and Zn(II)_2_Cys_6_ binuclear cluster DNA-binding motifs and regulate transcription of melanin biosynthesis genes in a developmentally specific manner. Mol Microbiol.

[21] Eliahu N, Igbaria A, Rose MS, Horwitz BA, Lev S (2007). Melanin biosynthesis in the maize pathogen *Cochliobolus heterostrophus* depends on two mitogen-activated protein kinases, Chk1 and Mps1, and the transcription factor Cmr1. Eukaryot Cell.

[22] Kihara J, Moriwaki A, Tanaka N, Tanaka C, Ueno M (2008). Characterization of the *BMR1* gene encoding a transcription factor for melanin biosynthesis genes in the phytopathogenic fungus *Bipolaris oryzae*. FEMS Microbiol Lett.

[23] Cho Y, Srivastava A, Ohm RA, Lawrence CB, Wang KH (2012). Transcription factor Amr1 induces melanin biosynthesis and suppresses virulence in *Alternaria brassicicola*. PLoS Pathog.

[24] Schumacher J (2016). DHN melanin biosynthesis in the plant pathogenic fungus *Botrytis cinerea* is based on two developmentally regulated key enzyme (PKS)-encoding genes. Mol Microbiol.

[25] Zhou Y, Yang L, Wu M, Chen W, Li G (2017). A single-nucleotide deletion in the transcription factor gene *bcsmr1* causes sclerotial-melanogenesis deficiency in *Botrytis cinerea*. Front Microbiol.

[26] Qin QM, Vallad GE, Wu BM, Subbarao KV (2006). Phylogenetic analyses of phytopathogenic isolates of *Verticillium* spp. Phytopathology.

[27] Klosterman SJ, Subbarao KV, Kang S, Veronese P, Gold SE (2011). Comparative genomics yields insights into niche adaptation of plant vascular wilt pathogens. PLoS Pathog.

[28] Paz Z, García-Pedrajas MD, Andrews DL, Klosterman SJ, Baeza-Montañez L (2011). One step construction of *Agrobacterium*-recombination-ready-plasmids (OSCAR), an efficient and robust tool for ATMT based gene deletion construction in fungi. Fungal Genet Biol.

[29] Dobinson KF, Grant SJ, Kang S (2004). Cloning and targeted disruption, via *Agrobacterium tumefaciens*-mediated transformation, of a trypsin protease gene from the vascular wilt fungus *Verticillium dahliae*. Curr Genet.

[30] Faino L, Seidl MF, Datema E, van den Berg GC, Janssen A (2015). Single-molecule real-time sequencing combined with optical mapping yields completely finished fungal genome. MBio.

[31] Kabir Z, Bhat RG, Subbarao KV (2004). Comparison of media for recovery of *Verticillium dahliae* from soil. Plant Disease.

[32] Pfaffl MW, Horgan GW, Dempfle L (2002). Relative expression software tool (REST) for group-wise comparison and statistical analysis of relative expression results in real-time PCR. Nucleic Acids Res.

[33] Cheng XX, Zhao LH, Klosterman SJ, Feng HJ, Feng ZL (2017). The endochitinase VDECH from *Verticillium dahliae* inhibits spore germination and activates plant defense responses. Plant Sci.

[34] Sarmiento-Villamil JL, García-Pedrajas NE, Baeza-Montañez L, García-Pedrajas MD (2018). The APSES transcription factor Vst1 is a key regulator of development in microsclerotium- and resting mycelium-producing *Verticillium* species. Mol Plant Pathol.

[35] Butler MJ, Day AW (1998). Fungal melanins: a review. Can J Microbiol.

[36] Brandt WH (1964). Morphogenesis in verticillium: effects of light and ultraviolet radiation on microsclerotia and melanin. Can J Bot.

[37] Hawke MA (1994). Studies on the Survival of Microsclerotia of Verticillium dahliae.

[38] Gessler NN, Egorova AS, Belozerskaya TA (2014). Melanin pigments of fungi under extreme environmental conditions (review). Appl Biochem Microbiol.

[39] Zhong Q, Gvozdenovic-Jeremic J, Webster P, Zhou J, Greenberg ML (2005). Loss of function of KRE5 suppresses temperature sensitivity of mutants lacking mitochondrial anionic lipids. Mol Biol Cell.

[40] Tamura K, Stecher G, Peterson D, Filipski A, Kumar S (2013). MEGA6: molecular evolutionary genetics analysis version 6.0. Mol Biol Evol.

[41] Tsuji G, Sugahara T, Fujii I, Mori Y, Ebizuka Y (2003). Evidence for involvement of two naphthol reductases in the first reduction step of melanin biosynthesis pathway of *Colletotrichum lagenarium*. Mycol Res.

[42] Perpetua NS, Kubo Y, Yasuda N, Takano Y, Furusawa I (1996). Cloning and characterization of a melanin biosynthetic THR1 reductase gene essential for appressorial penetration of *Colletotrichum lagenarium*. Mol Plant Microbe Interact.

[43] Kubo Y, Takano Y, Endo N, Yasuda N, Tajima S (1996). Cloning and structural analysis of the melanin biosynthesis gene SCD1 encoding scytalone dehydratase in *Colletotrichum lagenarium*. Appl Environ Microbiol.

[44] Fujii I, Yasuoka Y, Tsai HF, Chang YC, Kwon-Chung KJ (2004). Hydrolytic polyketide shortening by ayg1p, a novel enzyme involved in fungal melanin biosynthesis. J Biol Chem.

[45] Takano Y, Kubo Y, Shimizu K, Mise K, Okuno T (1995). Structural analysis of PKS1, a polyketide synthase gene involved in melanin biosynthesis in *Colletotrichum lagenarium*. Mol Gen Genet.

[46] Tsuji G, Fujikawa J, Ishida H, Horino O, Kubo Y (2001). Laccase gene *LAC1* of *Colletotrichum lagenarium* is not essential for melanin biosynthesis and pathogenicity. J Gen Plant Pathol.

[47] Wang G, Liu Z, Lin R, Li E, Mao Z (2016). Biosynthesis of antibiotic leucinostatins in bio-control fungus *Purpureocillium lilacinum* and their inhibition on *Phytophthora* revealed by genome mining. PLoS Pathog.

