# Corrigendum: Transcription factor VdCmr1 is required for pigment production, protection from UV irradiation, and regulates expression of melanin biosynthetic genes in *Verticillium dahliae*

**DOI:** 10.1099/mic.0.000661

**Published:** 2018-05-02

**Authors:** Yonglin Wang, Xiaoping Hu, Yulin Fang, Amy Anchieta, Polly H. Goldman, Gustavo Hernandez, Steven J. Klosterman

**Affiliations:** ^1^​ College of Forestry, Beijing Forestry University, Beijing, PR China; ^2^​ Department of Plant Pathology, College of Plant Protection, Northwest A&F University, Yangling, PR China; ^3^​ United States Department of Agriculture, Agricultural Research Service, 1636 E. Alisal St., Salinas, CA 93905, USA

**Keywords:** fungi, regulation, pigment, pathogenicity, DHN, melanin

There was an error in Fig. 6 of the published article. In the HOG-MAPK cascade the text ‘Ste11’ should be replaced by ‘Ssk2’. The corrected Fig. 6 is shown on the next page.

The author apologizes for any inconvenience caused.

**Fig. 6.**
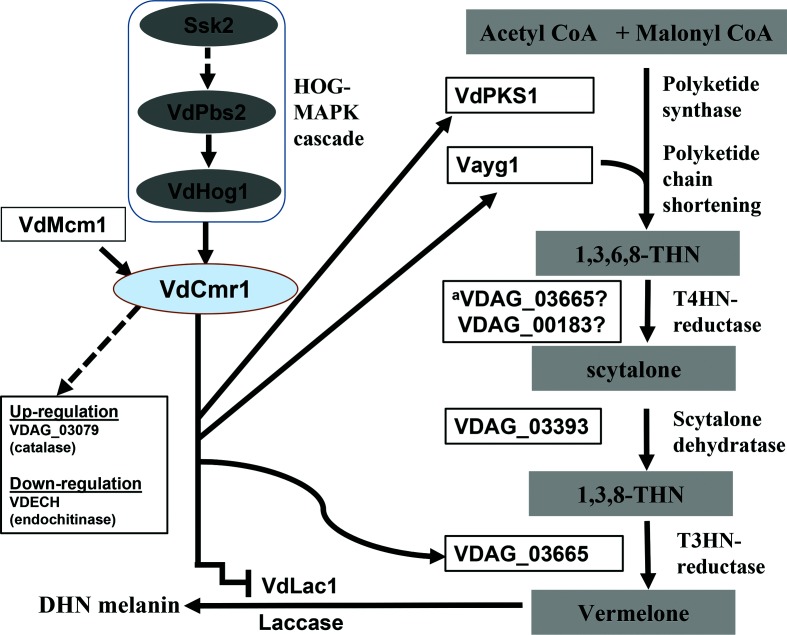
Schematic diagram depicting VdCmr1-dependent DHN melanogenesis in *V. dahliae*. Key proteins involving the DHN melanogenesis pathway are depicted from the initial acetyl-CoA or malonyl-CoA precursors through production of DHN melanin [4]. The Ssk2-Pbs2-Hog1 signalling cascade is a regulator of melanin biosynthesis in *V. dahliae* [14]. The transcription factor VdMcm1 and the polyketide chain shortening enzyme Vayg1 also modulate DHN melanogenesis. Our work identified several key genes that contribute to DHN melanin biosynthesis and that are regulated by VdCmr1. Some of these genes are found within the cluster enriched in DHN melanin biosynthetic genes within the *V. dahliae* genome. The non-melanin synthesis-related genes, up or downregulated during microsclerotial development [7], are regulated by VdCmr1 as demonstrated in this study; these genes VDAG_03079 (catalase) and VDAG_08741 VDECH (endochitinase, VDAG_08741) also reside outside of the gene cluster enriched in melanin biosynthetic genes. ^a^VDAG_03665 may catalyse both T4HN and T3HN reductase steps based upon the strong homology of VDAG_03665 to the THR1 protein of *Colletotrichum lagenarium*, which can catalyse both T4HN and T3HN reductase steps [41].

