# Supplementary material for: Transcription factor VdCmr1 is required for pigment production, protection from UV irradiation, and regulates expression of melanin biosynthetic genes in Verticillium dahliae
Source: Microbiology (Reading). 2018 Feb 27;164(4):685–96. doi: 10.1099/mic.0.000633 (PMC5982140; doi:10.1099/mic.0.000633)
Supplement: Supplementary File 1 [file mic-164-685-s001.pdf]

Supplementary Table 1. Oligonucleotide primer and probe sequences used in this study

**Primer/Probe name**	**Sequence (5’-> 3’)**	**function**
38F	GGGGACAGCTTTCTTGTACAAAGTGGAACCCAGCTTCTACCAGACTGC	VDAG_00189 5’-flank
1093R	GGGGACTGCTTTTTTGTACAAACTTGTGTTGAACGAAGGTCGCTTCC	
7F	GGGGACAACTTTGTATAGAAAAGTTGTTCAACGAGGTTGAAACTCAGG	VDAG_00189 3’-flank
1071R	GGGGACAACTTTGTATAATAAAGTTGTGCGTGCTACAGTCTCCTTCAA	
242F	ACGGTATCAACCAGGTCAGC	VDAG_00189 knockout screening
769R	TGCTGCGAAGTTAATGATGC	
Hyg-F	CCTGAACTCACCGCGACGTC	hygromycin gene-specific
Hyg-R	CTATCCTTTGCCCTCGGACGAGTG	
1905'F	GGGGACAGCTTTCTTGTACAAAGTGGAAAAATTATTACCGTCTGGCTCGTGT	VDAG_00190 5’-flank
1905'R	GGGGACTGCTTTTTTGTACAAACTTGTTTGAGCGACTGGTCTCCAAAGA	
1903'F	GGGGACAACTTTGTATAGAAAAGTTGTTACAAGAACACCACCCACTCC	VDAG_00190 3’-flank
1903'R	GGGGACAACTTTGTATAATAAAGTTGTGTACGAGCCTTTCCCTGTCG	
190F	CAAGACTGGTCCCTGTGCTAC	VDAG_00190 knockout screening
190R	CTTCGGCGATATGATTGAACT	
PL62	CAGGACCACCGTGGAACCAGT	VDAG_00195 5’-flank
PL695	GTCGTGACTGGGAAAACCCTGGCG AGCTCCCAGAGGGTTCCATTCA	
PL696	TCCTGTGTGAAATTGTTATCCGCT TGTGACACGCAGTGACTTCCGT	VDAG_00195 3’-flank
PL65	ACGACGTCAAGGTTGTCCACTA	
PL760	AGGAGCGGCGACAGGAAA	VDAG_00195 knockout screening
PL761	CGATGCGGCTGGTGAAAC	
Geneticinfor	CGCCAGGGTTTTCCCAGTCACGAC	geneticin resistant cassette
Geneticinrev	AGCGGATAACAATTTCACACAGGA	
UBIQ1	GCTCACCGGTAAGACTATCACA	RT-qPCR primer
UBIQ2	TTGGAAGTCGAATCATCC	TaqMan probe
UBIQ3	TTGGACTTCACATTGTCGATCGT	RT-qPCR primer
VDAG_00184-1	ACAAGCCGTTGTTCTATCACATCA	RT-qPCR primer
VDAG_00184-2	CAACCCCAAGCCCC	TaqMan probe

VDAG_00184-3	GCTGTCACCACGTCTGATAGATC	RT-qPCR primer
VDAG_00189-1	ACTGCTCAACGTTACGATGTTCTC	RT-qPCR primer
VDAG_00189-2	ATCGACCGCGACAACC	TaqMan probe
VDAG_00189-3	GCCACCAGGAAGGGATAGTTG	RT-qPCR primer
VDAG_00190-1	GCACGCCTGGTTTGCT	RT-qPCR primer
VDAG_00190-2	TCCATCGGGCTCCTTG	TaqMan probe
VDAG_00190-3	AATCTTTTTGCTCGACGCATCTTC	RT-qPCR primer
VDAG_00194/5-1	GCGATGGGTTCTCTTCCTCAAG	RT-qPCR primer
VDAG_00194/5-2	CAATGCCCACCTTTGC	TaqMan probe
VDAG_00194/5-3	GATCCGACGTTGAAGCAATGT	RT-qPCR primer
VDAG_00261-1	ACGGCCTCGTCGACAAG	RT-qPCR primer
VDAG_00261-2	TCGCGGCCCTTGTTG	TaqMan probe
VDAG_00261-3	GTAAAGGTCATGAGGGTGGTGAAG	RT-qPCR primer
VDAG_03079-1	TCGGCCTCGTGCAGTAC	RT-qPCR primer
VDAG_03079-2	ATCCAGGCCAAGTACC	TaqMan probe
VDAG_03079-3	CGGGCGAGATGTTGTAAAGCT	RT-qPCR primer
VDAG_04954-1	CCTGTGGGAGACCAAATGGA	RT-qPCR primer
VDAG_04954-2	TTGGTGCATGGAAATC	TaqMan probe
VDAG_04954-3	CGTCATGGAAAGGGTACACACTT	RT-qPCR primer
VDAG_08741-1	CCAAGCGGCGGTTACG	RT-qPCR primer
VDAG_08741-2	CTGCCCGGCCATTTG	TaqMan probe
VDAG_08741-3	CTGCTGGGACCGTTCCA	RT-qPCR primer
VDAG_00192-1	GCCGGACAAGGCAATATGC	RT-qPCR primer
VDAG_00192-2	CTCGCCCCTGTGCTGC	TaqMan probe
VDAG_00192-3	CCAATGTATAGGCACCAAGACTGAT	RT-qPCR primer
VDAG_03665-1	CCGATGACGAAGTCGACGAATAC	RT-qPCR primer
VDAG_03665-2	CCGACACGGTGAACTG	TaqMan probe
VDAG_03665-3	ACGCGAGCGATGTCGAT	RT-qPCR primer
VDAG_05181-1	CGGCCTGCCCATCGA	RT-qPCR primer
VDAG_05181-2	ATTGCCCGCGTTGTTT	TaqMan probe
VDAG_05181-3	TCCTGGGAGGCGAGGAA	RT-qPCR primer

VdCMRPF	ACTGCCGCTGTGACACGC	Primer for VdCmr1 Southern probe
VdCMRPR	AACGCTGGACCCTGGCCT	Primer for VdCmr1 Southern probe
SP190-F	TCCTTATCGACTCCCCTTGC	Primer for VdPKS1 Southern probe
SP190-R	TTGGATGAGCTTGTGGACCA	Primer for VdPKS1 Southern probe
Hyg- F	GCCGATGCAAAGTGCCGATAAACA	Primer for VdLac1 Southern probe
OSC-R	CGCCAATATATCCTGTCAAACACT	Primer for VdLac1 Southern probe
VdCmr1-comF	GTCGCCTGAGAATCCGAGTA	Mutant complementation
VdCmr1-comR	TCACCAACACCCCCAGCCGCA	Mutant complementation
VdCmr1-up	ACGGCACCCAAAGACCATAG	RT-PCR of VdCmr1
VdCmr1-down	TCAACGACACCAATGCCGTA	RT-PCR of VdCmr1
Hyg477F	GATGTTGGCGACCTCGTATT	Hygromycin resistance gene confirmation primer
Hyg1055R	GATGTAGGAGGGCGTGGATA	Hygromycin resistance gene confirmation primer

Supplementary Figure 1

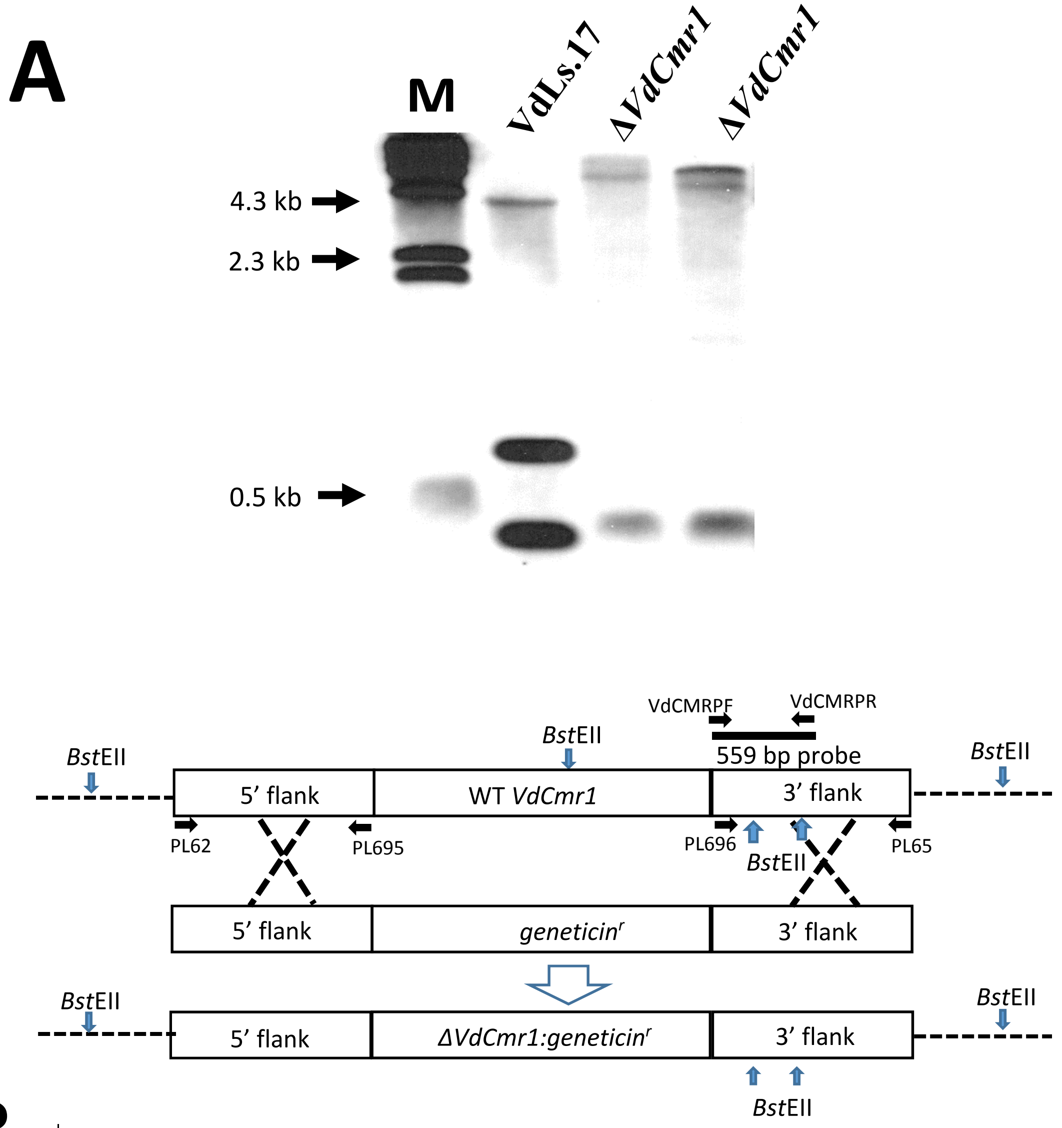

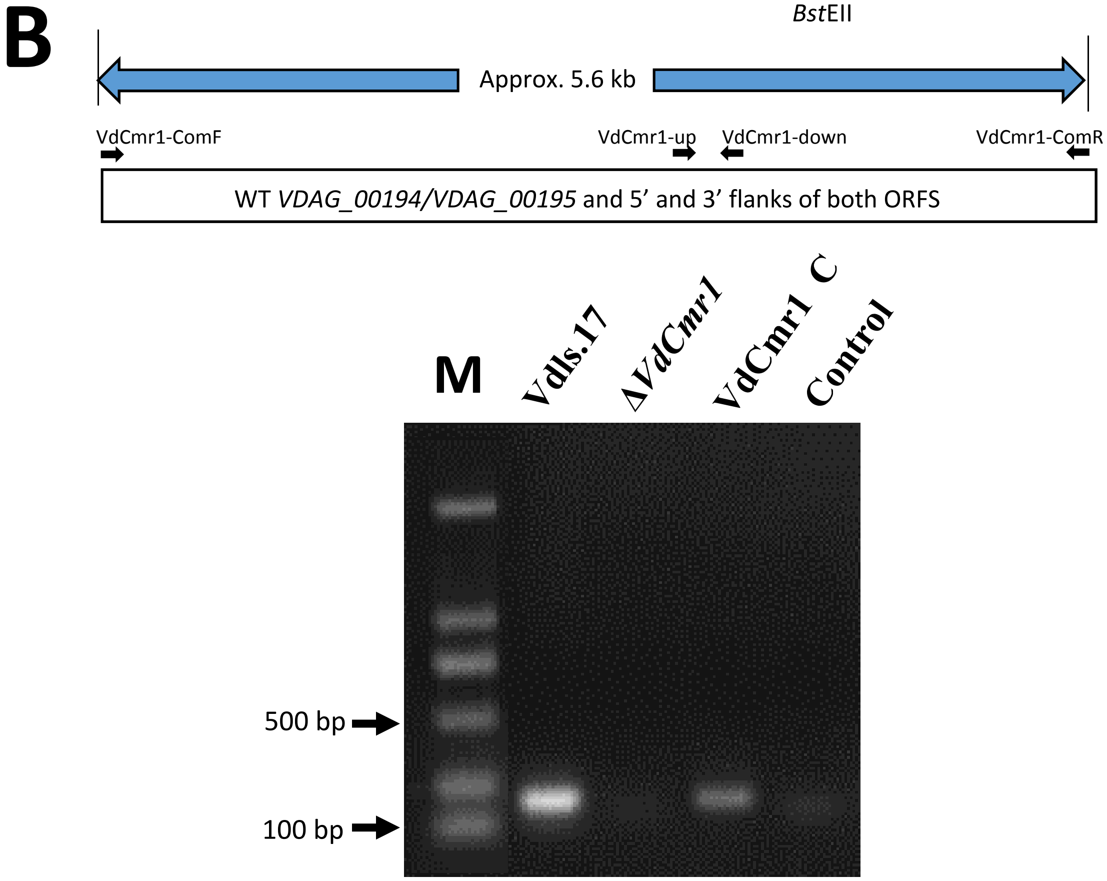

Supplementary Fig. 1 Confirmation of the *VdCmr1* knockout mutant and its subsequent complementation. **A)** The primer pair VdCMRPF and VdCMRPR (Supplementary Table 1) were used to generate the 559 bp DIG-labeled probe for confirmation of the *VdCmr1* mutation. The *VdCmr1* gene replacement was confirmed by using a digest (NEB), in NEB buffer 3.1, with 2 µl enzyme, 2.5 µg DNA, and incubated at 60ºC for 4 hrs. Probe hybridization of the *Bste*II-digested DNA was expected to yield bands of nearly 390 bp, 850 bp and 4500 bp for the wild type, but the 850 bp and 4500 bp bands are absent in the mutant, as shown in Fig. S1A. Rather, a band much larger than 4500 bp was expected in the Δ*VdCmr1*-digested DNA because the insertion of geneticin resistance marker removed the *Bst*EII site in the wild type ORF, yet the probe still bound the larger fragments and the smaller fragment at about 390 bp as shown in Fig. S1A. The dashed lines in the "X" pattern refer to regions of homologous recombination. **B)** For Δ*VdCmr1* complementation, a fragment of approx. 5.6 kb containing the *VdCmr1*-coding region, including *VDAG_00194* (because these were previously erroneously annotated as two separate genes; [8]) was amplified from genomic DNA with primers VdCmr1-comF/R (Table S1). This fragment was co-transformed into protoplasts of the *VdCmr1* mutant along with a gene encoding *hygromycin* resistance from pRF-HU (Fungal Genetics Stock Center, Kansas City, Missouri). The complementated strains were selected on hygromycin B-containing PDA medium and confirmed by reverse transcription PCR (RT-PCR) as shown in Fig. S1B, using primer pairs VdCmr1-up/down (Table S1). The 191 bp RT-PCR product was amplified using the primers VdCmr1-up and –down (Table S1). WT = wild type. Drawing not to scale.

Supplemental Figure 2

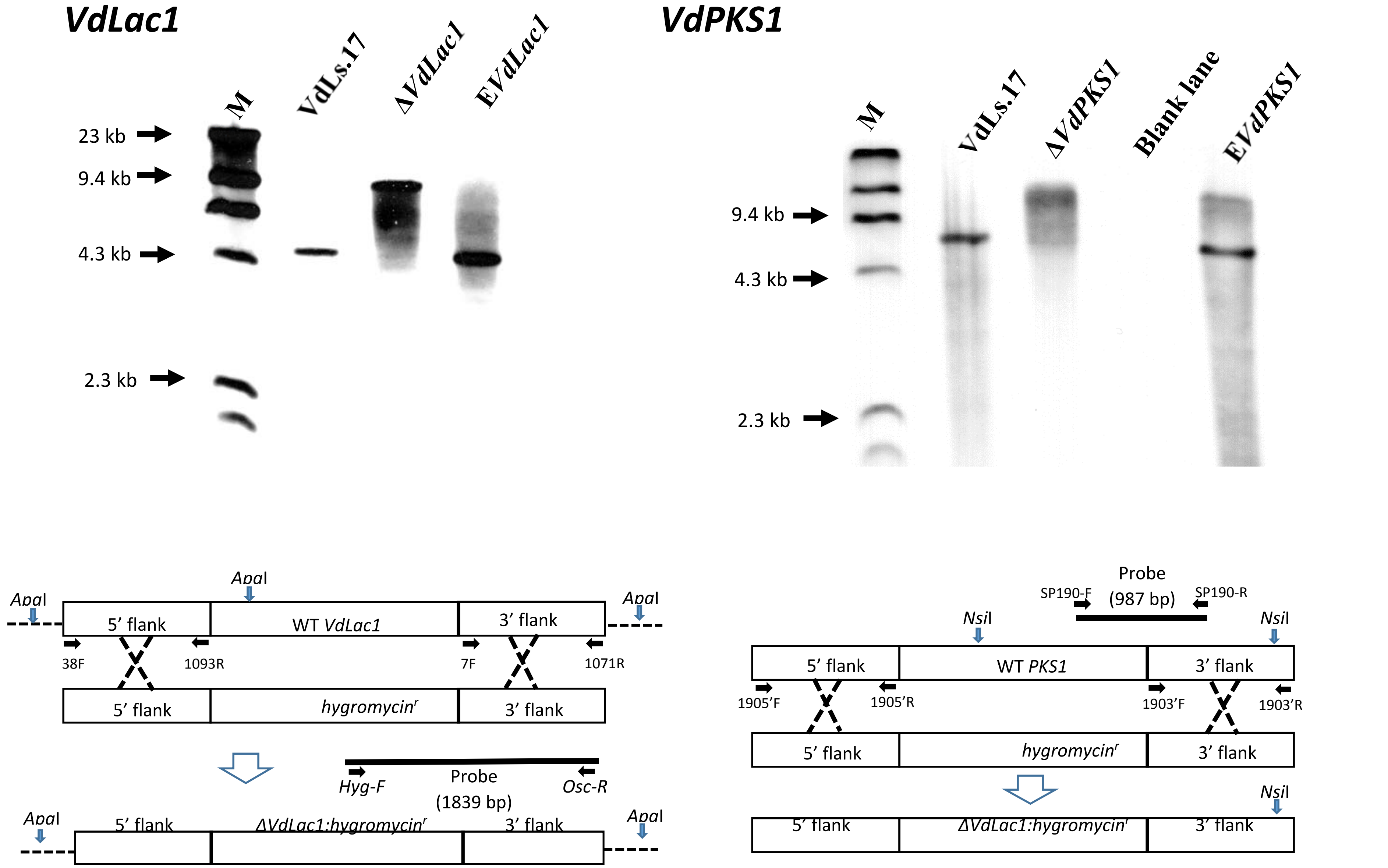

Supplementary Fig. 2 Confirmation of the *VdPKS1* and *VdLac1* knockout mutants. The Southern probes for the wild type (WT) gene replacements were prepared using Hyg-F and Osc-R primers for *VdLac1* and primers SP190F and SP190R for the *VdPKS1* probe. For the confirmation of the *VdLac1* gene replacement, the DNA was *Apa*I (NEB)-digested in CutSmart® Buffer (NEB), 2.5 µl enzyme, 2 µg DNA, and 25ºC overnight. PCR-50 uL reactions. As shown in the schematic below the blot, the anticipated digestion product for the wild type and ectopic DNA was a 5890 bp fragment, and a 9541 bp fragment for the *VdLac1* gene replacement mutant strain, since the *Apa*I site is removed by the *hygromycin* resistance gene in the mutant strain. In actuality the digested fragments were all three strains were about 1500 bp shorter than those expected sizes, presumably due to missassembly near the *VdLac1* coding region in the originally annotated genome [28]. Confirmation of the *VdPKS1* gene replacement with the depicted hybridization probe was carried out using *Nsi*I-HF (NEB) digests, where a band of 5556 bp was expected for the VdLs.17 (wild type), and only a band of larger size was expected in the knockout strains because the enzyme cuts once in the transformed DNA, within the region for which the probe binds. Dashed lines in the "X" pattern refer to regions of homologous recombination. Drawings not to scale.

Supplementary figure 3

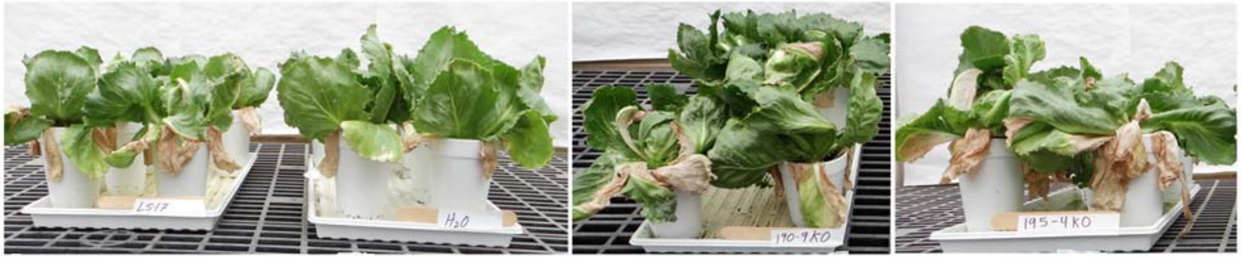

VdLs.17 Water Δ*VdPKS1* Δ*VdCmr1*

Supplementary Fig. 3 Pathogenicity assay on lettuce.

Lettuce seedlings inoculated with wild-type strain VdLs17, the VdPKS1 deletion mutant (7KO), the VdCmr1 deletion mutant, or a mock-inoculated (water) control.
